# Host-Orf virus interactions: A comprehensive review of pathogenesis and immune evasion mechanisms

**DOI:** 10.1080/21505594.2026.2682640

**Published:** 2026-07-31

**Authors:** Feng Pang, Shaobo Liang, Jingjin Hu, Weijie Zhou, Xiaoyue Wang, Zhentao Cheng

**Affiliations:** Department of Veterinary Medicine, College of Animal Science, Guizhou University, Guiyang, China

**Keywords:** Orf virus, pathogenesis, immune evasion, immunomodulatory proteins, virus-host interactions

## Abstract

Orf virus (ORFV), a zoonotic parapoxvirus, causes contagious ecthyma in small ruminants. It is uniquely characterized by its ability to cause proliferative, self-resolving skin lesions and systematically evade host immunity to cause repeated infections. This review provides an in-depth analysis of ORFV pathogenesis, focusing on viral entry, replication, and lesion development. Furthermore, we highlight the sophisticated immune evasion strategies employed by ORFV that enable it to thrive as a pathogen. By integrating recent molecular and immunological advances, we illustrate how key viral immunomodulatory proteins systematically subvert innate and adaptive host defenses, including interferon pathways, NF-κB signaling, and apoptosis. Deciphering these complex virus-host interactions not only clarifies ORFV’s evolutionary success but is also critical for designing effective vaccines and novel therapeutics.

## Introduction

Orf virus (ORFV) is an epitheliotropic member of the *Parapoxvirus* genus (*Poxviridae* family) and is the etiological agent of contagious ecthyma. This globally distributed virus primarily affects small ruminants, leading to painful, proliferative lesions on their skin and mucous membranes, and poses a notable zoonotic risk [1–3]. In natural hosts such as sheep and goats, ORFV induces a characteristic clinical course marked by a rapid progression of lesions through macular, papular, vesicular, pustular, and finally scab stages, typically healing within 4–6 weeks [4,5]. Crucially, ORFV is a zoonotic pathogen that transmits to humans via direct contact or exposure to contaminated fomites. While rarely fatal in adult animals, ORFV infection causes significant economic losses resulting from weight loss in lambs, reduced milk production in ewes, and predisposition to secondary bacterial infections [6].

A remarkable feature of ORFV is its ability to cause reinfection in previously exposed hosts, indicating a failure to establish robust, long-lasting sterilizing immunity. This clinical observation confirms that ORFV employs sophisticated strategies to subvert host immunity [7–9]. Understanding its pathogenesis and immune evasion mechanisms is fundamental to the design of novel therapeutics and vaccines.

## Viral structure and genome

Parapoxviruses are distinguished from other poxviruses by their ovoid morphology, smaller size (e.g. 260 × 160 nm for ORFV), and high G+C content of around 63% [10,11]. Their most diagnostic feature is a unique spiral (criss-cross) tubule-like surface pattern on the viral coat. ORFV, like other poxviruses, replicates within the host cell’s cytoplasm using its own replication machinery. It also produces two principal virion forms. Mature virions (MV) represent the most abundant and stable infectious form of ORFV. They are released following host cell lysis and can remain infectious in the extracellular environment [12,13]. In contrast, enveloped virions (EV) acquire an additional host-derived lipid envelope from membranes of the Golgi apparatus. This envelope equips EV for efficient cell-to-cell spread, long-range dissemination, and immune evasion within the host.

**Figure 1.**
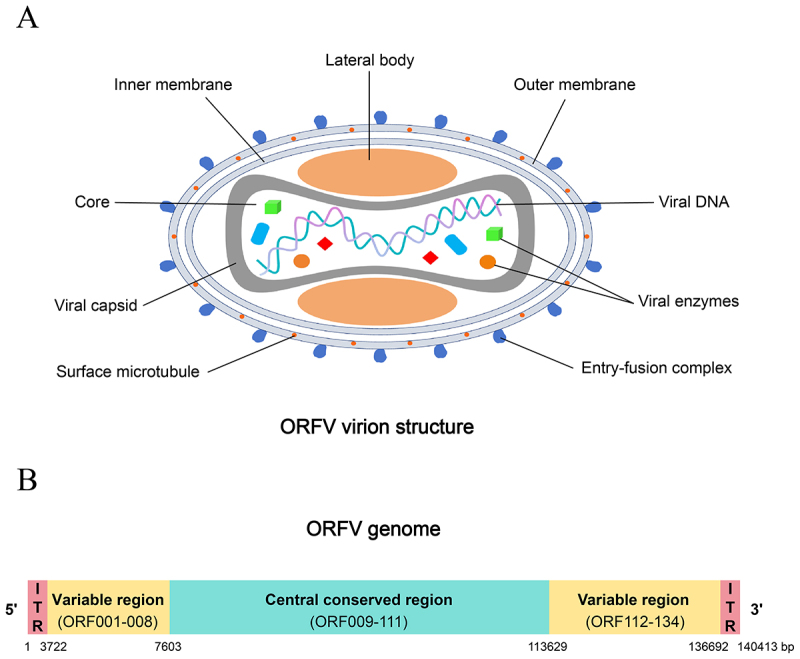
Schematic representations of virion structure and genomic organization of ORFV. (A) ORFV virion structure. ORFV is a smaller, ovoid poxvirus with a distinctive spiral surface pattern. It replicates in the cytoplasm and produces two major infectious forms: the most abundant and stable mature virion (MV) and the enveloped virion (EV), which has an additional membrane. The virion is composed of a DNA genome within a dumbbell-shaped core, surrounded by nucleocapsids, lateral bodies, and a double membrane with surface proteins. (B) Genomic organization of ORFV. The ORFV genome is a linear double-stranded DNA molecule (134–140 kb) with inverted terminal repeats (ITRs) and covalently closed hairpin ends. Its central conserved core (ORFs 009–111) harbors genes essential for replication and virion assembly, whereas the flanking variable regions (ORFs 001–008, 112–134) are enriched for immunomodulatory and virulence factors. While the ORF numbering extends to 134 for historical reasons, the ORFV genome actually encodes 132 predicted genes. Nucleotide coordinates at positions 1, 3722, 7603, 113,629, 136,692, and 140,413 bp correspond to the ORFV-SY17 strain (GenBank accession number: MG712417).

ORFV’s double-stranded DNA genome, spanning 134–140 kb and encoding 132 open reading frames (ORFs), is organized into a conserved central core (ORFs 009–111) and variable terminal segments (ORFs 001–008, 112–134) [14–19](Figure 1). The central region accounts for approximately 80% of the whole genome and contains genes critical for viral replication and virion assembly. The terminal regions encode genes involved in virulence, pathogenesis, immunomodulation and host range. The genome terminates in ~ 3 kb inverted terminal repeats (ITRs), and each end is sealed by a 100 bp hairpin loop that covalently closes the DNA strands. The B2L gene (ORFV011) encodes a highly immunogenic ~42 kDa envelope protein, homologous to vaccinia virus p37K (F13L) protein. Functionally, B2L is essential for generating the EV virion form. It orchestrates the wrapping of intracellular mature virions and their subsequent transport to the cell surface for egress [20–22]. The F1L gene (ORFV059) encodes another highly conserved, late-expressed ~39 kDa structural envelope protein. F1L is essential for viral assembly and morphogenesis to ensure virion stability and infectivity [23,24]. B2L and F1L are the most conserved proteins among parapoxviruses and are extensively employed as molecular markers for ORFV phylogenetic studies [25–29]. As the major immunodominant antigens of ORFV, B2L and F1L provoke potent humoral and cellular immune responses in infected hosts. Consequently, these immunogenic properties make them key targets for serological diagnosis and vaccine development [30–36].

Genomic studies have elucidated the genetic makeup of ORFV, revealing a large, dsDNA genome encoding numerous immunomodulatory proteins, such as GIF, VEGF-E, and vIL-10 [37]. Comparing genomes from different geographic locations or outbreaks helps track viral evolution and identify genes under selective pressure, often pointing toward novel immunomodulators, virulence factors and host range determinants [38–44].

## Pathogenesis of ORFV

### Viral attachment, entry and initial replication

ORFV transmission occurs through direct contact or via fomites contaminated with scab material, where the virus can remain viable for months [45]. ORFV infection is generally facilitated by breaches in the skin or mucosal barrier, such as abrasions, scratches, or wounds. However, the absolute necessity of preexisting skin injury remains a subject of ongoing investigation. Evidence suggests that infection may also occur through apparently intact skin or via alternative routes, including contact with infected milk [46,47]. Unlike many viruses that require specific receptors, ORFV likely mediates cellular attachment through its F1L envelope protein, which specifically interacts with general cell surface molecule heparan sulfate proteoglycans (HSPGs) [48]. ORFV enters target cells primarily through clathrin-mediated endocytosis and macropinocytosis, with a minor contribution from caveolae-dependent endocytosis. This entry process is both dynamin-dependent and requires an acidic environment [49]. KCNE4 plays a critical role in ORFV infection by facilitating viral entry into the target cells [50]. While the Vaccinia virus (VACV) L1 envelope protein is essential for viral entry by binding specific cell surface receptors distinct from proteoglycans [51], the role of its ORFV homolog, ORFV047, is not yet defined, although interactions with host proteins SERP1 and PABPC4 have been identified [52].

Upon entry, ORFV primarily targets epidermal keratinocytes and dermal fibroblasts in the oral mucosa [53,54]. Like all poxviruses, its entire replication cycle occurs within the cytoplasm of the host cell, utilizing virus-encoded DNA polymerase and other replication machinery [4]. Early gene expression occurs within hours, producing proteins necessary for DNA synthesis and immune modulation. Intermediate and late genes follow, encoding structural proteins for virion assembly.

### Lesion development and histopathology

The hallmark of ORFV pathogenesis is the development of proliferative, scab-forming lesions. In natural hosts such as sheep and goats, the disease follows a characteristic clinical course [54,55]: initial papules appear 3–7 days post-infection, then progress through vesicular and pustular stages to form thick, brownish scabs over 2–4 weeks. Lesions typically localize on the lips, nostrils, oral mucosa, and occasionally on the coronary bands or teats. The entire cycle, from infection to the shedding of the scab (which may leave a small scar), typically resolves within 4–6 weeks in uncomplicated cases. In immunocompetent hosts, the self-limiting nature of ORFV infection results from coordinated innate and adaptive immunity. However, in immunocompromised hosts, the virus can cause extensive, non-healing lesions and, disseminated infection. ORFV can persist in scabs for months, serving as a reservoir for transmission. Due to skin barrier disruption, secondary bacterial infections are common and can exacerbate morbidity. ORFV infection is generally considered localized, with systemic spread thought to be rare. Nevertheless, recent studies [47,56] have challenged this notion by detecting viremia as well as viral shedding in blood, saliva, and milk, indicating a more intricate pathogenesis.

Histological examination of ORFV lesions reveals virus-driven epidermal changes, including acanthosis, elongation of rete ridges, and pseudoepitheliomatous hyperplasia, accompanied by a prominent inflammatory cell infiltrate [57,58]. These proliferative and vascular alterations are largely attributable to viral virulence factors such as the ORFV-encoded VEGF-E, which induces angiogenesis and increases vascular permeability, thereby supporting tissue expansion and local viral dissemination.

The disease manifestations and lesion characteristics vary significantly depending on the host species and their immune status. In humans, who act as accidental or dead-end hosts, zoonotic ORFV infection typically presents as a solitary, localized lesion on the hands, fingers and forearms-the primary points of contact with infected animals [59–63]. The lesion typically starts as a small, firm, red-to-blue papule. It then evolves into a hemorrhagic pustule or blood-filled blister (bulla), develops a central crust, transforms into a weeping nodule, and finally becomes coated with a thick, dry crust before healing. While the disease is self-limiting (healing in 3 to 8 weeks) in healthy individuals, immunosuppressed patients may develop progressive, non-healing, tumor-like lesions exceeding 5 cm in diameter and characterized by extensive vascularization.

### Viral-driven angiogenesis

Beyond simply evading immunity, ORFV encodes factors that actively drive the formation of its characteristic skin lesions. Notably, the virus encodes a potent secreted homolog of vascular endothelial growth factor (VEGF), VEGF-E (ORFV132)-a defining feature of parapoxviruses [64,65]. VEGF-E signals through VEGFR-2 and neuropilin-1 but not VEGFR-1 or VEGFR-3, distinguishing it from all known mammalian VEGF family members. This triggers a signaling cascade that promotes endothelial cell proliferation (angiogenesis) and increases vascular permeability. The intense proliferation of endothelial cells and neighboring keratinocytes contributes to the proliferative nature of the Orf lesion [66]. This highly vascularized and swollen tissue provides a nutrient-rich, immune-privileged microenvironment ideal for high-titer viral replication and protects the virus from systemic immune surveillance. The VEGF-E-driven unique vascular pathology largely dictates ORFV’s clinical presentation and virulence [67], with ORFV132 knockout causing greater attenuation than ORFV112 knockout in a goat model [68]. Recent evidence further reveals that ORFV-driven angiogenesis is accompanied by a tumor-promoting inflammatory response [69]. In naturally infected sheep and goats, ORFV lesions show marked infiltration of CD163^+^ macrophages that co-express EGFR and VEGFR2, along with overexpression of IL-6. This suggests that viral VEGF-E signaling synergizes with EGFR-expressing macrophages to create a pro-tumor microenvironment, highlighting a novel oncogenic-like mechanism beyond simple vascular proliferation.

ORFV VEGF-E exhibits significant sequence polymorphism, which can be broadly classified into two major genotypes, NZ2-like and NZ7-like, sharing only ~41% amino acid identity. However, key functional domains such as the cystine-knot motif and the VEGFR-2 binding site remain highly conserved across isolates [70]. In recent years, NZ7-like variants have become the predominant circulating strains in Asian countries, including China and India [71,72]. Despite this sequence and structural heterogeneity, it does not fundamentally alter VEGF-E’s angiogenic function, but rather reflects geographic adaptation and potential host species preference. For instance, NZ7-like viruses appear to be more frequently associated with goats than with sheep. Overall, VEGF-E diversity contributes more to viral epidemiology and host adaptation than to intrinsic virulence.

## Host immune response to ORFV

### Cellular immune response

The immune response to ORFV is complex, involving both humoral and cellular components, but these arms of immunity play distinct roles in the control and resolution of infection. The local cellular immune response within cutaneous lesions is considered the primary defense mechanism against ORFV infection. Histological analysis of skin from primarily infected and reinfected sheep shows that various immune cells are activated, and a large number of neutrophils, dendritic cells (DCs), B cells, and T cells are continuously recruited to the site of infection. In both primary and reinfection lesions, CD4^+^ T cells and DCs accumulate faster than other cell types, with CD4^+^ T cells representing the predominant T lymphocytes in the skin [73,74]. Concurrently, ORFV infection induces the massive release of various cytokines such as interleukin-1β (IL-1β), IL-8, IL-2, GM-CSF, TNF-α, and IFN-γ [53,75,76].

The depletion of CD4^+^ T cells in sheep prevents both the clearance of ORFV from the skin and the production of antibodies following infection, indicating that CD4^+^ T cells are indispensable for orchestrating anti-ORFV immunity [77]. Cyclosporin A (CsA) treatment abolished protective immunity in lambs reinfected with ORFV, resulting in severe, persistent lesions resembling primary infection [78]. Mechanistically, CsA suppresses the recruitment of key immune cells (CD4^+^ T cells, B cells, DCs) to the lesion site and critically inhibits the production of Th-1 specific IL-2 and IFN-γ.

### Humoral immune response

Infection with ORFV consistently induces a detectable humoral immune response in sheep [79,80]. However, the protective role of these antibodies remains controversial. Serological evaluations reveal that all infected animals produce virus-specific antibodies, particularly against a 40 kDa surface tubule protein, but antibody titers did not correlate with lesion severity or duration [79]. Similarly, Koptopoulos et al. demonstrated the presence of complement-dependent cytotoxic antibodies in experimentally infected sheep, yet naturally infected animals rarely showed such antibodies, and no correlation with protection was observed [80]. Researchers further showed that passively acquired maternal antibodies via colostrum failed to protect lambs from subsequent ORFV challenge, whereas vaccination induced protection associated with delayed-type hypersensitivity rather than antibody levels [81].

However, a subsequent *in vivo* study utilizing T-cell subset depletion [77] provided a more nuanced perspective, highlighting the critical importance of humoral immunity. It is now understood that high preexisting or rapidly induced antibody titers containing neutralizing and cytolytic activities, are strongly associated with the rapid resolution of severe lesions. While cellular immunity is indispensable, specific antibodies working in synergy with CD4^+^ T-cells are crucial for neutralizing free virions, destroying infected keratinocytes, and ultimately clearing ORFV from the host’s skin. In summary, ORFV induces a functional, neutralizing humoral response that, while insufficient for complete protection on its own, serves as a vital component of the host’s coordinated antiviral defense.

### Host-specific immune contexts and zoonotic implications

The clinical manifestations and resolution of ORFV lesions are heavily dictated by the host’s specific immune context. In natural hosts, such as sheep and goats, prolonged co-evolution has resulted in a host-pathogen equilibrium where ORFV effectively subverts sterilizing immunity, leading to recurrent infections with typical proliferative lesions. Conversely, in humans, an accidental, dead-end host, ORFV infection is generally restricted to solitary, self-resolving lesions. This suggests that the human innate and adaptive immune machinery, including species-specific differences in PRR affinities and interferon sensitivities, may be more resilient against ORFV’s immunomodulatory arsenal. The critical role of host immune competence is starkly highlighted in zoonotic infections of immunocompromised humans, where ORFV causes severe, giant, and non-healing progressive lesions due to impaired T-cell and macrophage functions. Therefore, ORFV’s virulence is not an absolute viral trait but is highly contextual, depending on whether the viral immune evasion proteins can successfully outmaneuver the specific host species’ immune pathways.

### Multi-omics profiling of host responses

Beyond classical cellular and humoral immunity, ORFV also triggers extensive transcriptomic reprogramming. RNA-seq analyses have revealed dynamic viral modulation of host immune, inflammatory, and apoptotic pathways in a wide range of host cells [82,83]. Furthermore, post-transcriptional epigenetic-like regulation via non-coding RNAs is pivotal. ORFV early infection reprograms the host circular RNA (circRNA) landscape, proposing a network where circRNAs act as miRNA sponges to influence inflammation and cell migration [84]. Specifically, ORFV exploits host miRNAs, such as the cfa-let-7a/THBS1 axis, which may suppress apoptosis and facilitate viral persistence [85].

Mass spectrometry-based proteomics and metabolomics further reveal how these upstream regulations are ultimately executed to reshape the cellular environment. Proteomic profiling underscores viral-induced post-transcriptional and translational regulation. For instance, ORFV potently activates bone marrow-derived dendritic cells (BMDCs), significantly altering the expression of key proteins implicated in antigen presentation, immune response, cell adhesion, and apoptosis regulation [86]. In infected GSF cells, widespread proteomic changes successfully identified the heat shock protein HSPA1B as a novel host restriction factor that actively inhibits ORFV replication during the middle and late infection phases [87]. Integrated non-targeted metabolomic and proteomic analyses demonstrate that early ORFV infection in OFTU cells significantly alters hundreds of host molecules, heavily skewing lipid, amino acid, nucleotide, and glucose metabolic networks [88]. Crucially, ORFV replication exhibits a strict dependence on host glucose metabolism and de novo fatty acid synthesis, while remaining independent of glutamine. Collectively, integrating classical epigenetic mechanisms with multi-omics (transcriptomic, proteomic, and metabolomic) profiling provides a highly comprehensive understanding of the ORFV-host dynamic interplay. Future functional studies are needed to validate these connections.

## Viral immune evasion mechanisms

ORFV’s capacity to reinfect hosts and cause persistent lesions is a direct consequence of its sophisticated arsenal of immunomodulatory proteins. These virulence factors form a multi-layered defense strategy, systematically subverting both innate and adaptive responses.

### Evasion of innate immunity

The innate immune system acts as the first line of defense against viruses by using pattern recognition receptors (PRRs) to detect pathogen-associated molecular patterns (PAMPs). This recognition rapidly triggers the production of antiviral effectors, primarily interferons (IFNs) and pro-inflammatory cytokines [89,90]. In response, ORFV utilizes a sophisticated multi-pronged strategy to disrupt this initial signaling and neutralize the host’s early antiviral efforts.

#### Inhibition of the interferon (IFN) pathway

Interferons (IFNs), particularly type I IFNs such as IFN-α/β, play a pivotal role in establishing a cellular antiviral state. Following engagement with their cognate receptor (IFNAR), these cytokines activate the JAK/STAT signaling cascade, resulting in the phosphorylation of STAT1 and STAT2. The subsequent nuclear translocation of this activated complex drives the transcription of hundreds of IFN-stimulated genes (ISGs), which work synergistically to suppress viral replication [91,92].

ORFV employs multiple strategies to antagonize this critical antiviral defense. First, the virus blocks IFN production at the transcriptional level. ORFV inhibits dsRNA-induced IFN-β expression in HEK293 cells by disrupting RIG-I-dependent signaling through the ORFV020 protein [93]. Similarly, ORFV strongly inhibits dsDNA-triggered IFN-β expression through both STING-dependent (in human dermal fibroblasts and THP-1 cells) and STING-independent (in STING/TLR-deficient HEK293 cells) pathways [94].

Second, ORFV interferes with IFN signaling downstream of receptor engagement. In infected HeLa cells, ORFV suppressed the JAK/STAT pathway by dephosphorylating STAT1 at Tyr701, thereby almost completely abolishing ISG expression induced by both IFN-α and IFN-γ. Although ORFV057 is implicated, definitive validation of its role in disrupting JAK/STAT signaling through functional analyses is still needed [95]. Deletion of ORFV116 resulted in elevated expression of ISGs and the pro-inflammatory cytokine IL-8 in HeLa cells compared to wild-type virus, suggesting that ORFV116 contributes to the suppression of host interferon responses and inflammation [96].

Third, ORFV disrupts downstream antiviral effectors such as dsRNA-dependent protein kinase (PKR). Viral dsRNA activates host PKR, which phosphorylates eukaryotic initiation factor 2α (eIF2α) to halt global protein synthesis and restrict viral replication [97,98]. ORFV020, a functional homolog of vaccinia virus E3L [99,100] exists as two isoforms, the full-length ORFV020 and the N-terminally truncated sh20. Although they exhibit different subcellular localizations, both directly bind to PKR and its activator dsRNA, leading to PKR inactivation [101]. ORFV020 also binds the PKR activator PACT, inhibiting the PKR-PACT interaction and further suppressing PKR activation [102]. Additionally, ORFV020 disrupts the RNA-editing function of ADAR1, and repurposes it to inhibit PKR and amplify viral immune evasion. Meanwhile, ADAR1 enhances ORFV replication by suppressing PKR and stabilizing ORFV020 expression [103].

**Figure 2.**
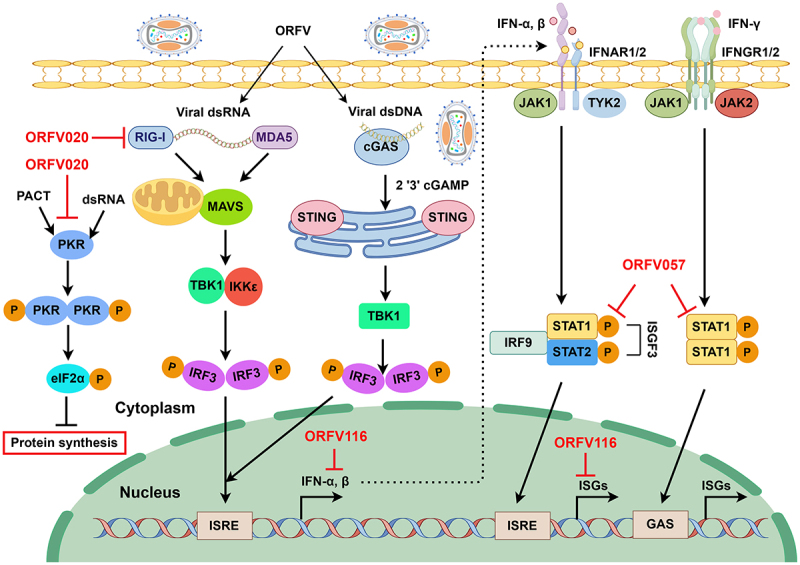
Multifaceted strategies of ORFV to evade host interferon and ISG responses. ORFV employs multiple viral proteins to antagonize innate antiviral signaling. ORFV020 inhibits IFN-β production by disrupting RIG-I signaling and inactivates PKR to maintain protein synthesis. The virus also suppresses the JAK/STAT pathway, potentially via ORFV057, to block ISGs expression, and the ORFV116 protein helps dampen interferon and inflammatory responses. Furthermore, ORFV inhibits IFN-β induction by dsDNA through both STING-dependent and independent mechanisms.

Finally, genetic heterogeneity may influence the efficacy of these antagonistic mechanisms. Alignment analyses of ORFV020 from various sheep and goat isolates reveal species-specific clustering and genetic diversity among parapoxviruses [104]. This structural variation in the ORFV20 protein suggests that different ORFV strains may possess varying efficiencies in antagonizing the host interferon response. Multifaceted strategies employed by ORFV to evade host interferon and ISG responses are illustrated in Figure 2.

#### Modulation of cytokines and chemokines

The anti-inflammatory cytokine interleukin-10 (IL-10) naturally mitigates inflammation and prevents immunopathology by suppressing pro-inflammatory cytokines and impairing the antigen-presenting function of dendritic cells (DCs) and macrophages, thereby halting T-cell activation [105–107]. To control the local inflammatory environment and leukocyte trafficking, ORFV encodes secreted proteins that mimic or neutralize host cytokines and chemokines. A primary strategy involves ORFV127, an early gene which encodes vIL-10, a viral homolog of the host anti-inflammatory cytokine interleukin-10 (IL-10) [108,109]. Mimicking its cellular counterpart, vIL-10 suppresses the production of IFN-γ and IL-2 by T-cells and NK cells [110]. Furthermore, vIL-10 inhibits the maturation, antigen-presenting capacity, and migration of murine and human DCs [111,112]. *In vivo*, it impairs the recruitment of monocytes, DCs, and mast cells to inflamed skin and blocks the trafficking of DCs from the skin to draining lymph nodes [113]. In activated human THP-1 monocytes, vIL-10 primarily suppresses cytokine synthesis, though it only partially inhibits cellular proliferation [114]. Further studies demonstrated that vIL-10 is a virulence factor contributing to the pathogenesis of ORFV in sheep [109,115].

Another critical immunomodulator is the Granulocyte-Macrophage Colony-Stimulating Factor (GM-CSF) and IL-2 Inhibitory Factor (GIF), a secreted protein encoded by the intermediate-late viral gene ORFV117 [116,117]. GIF exhibits a dual inhibitory function by binding and neutralizing both GM-CSF and IL-2. The inhibition of GM-CSF impairs the differentiation and activation of key antigen-presenting cells. Concurrently, the neutralization of IL-2, a critical T-cell growth factor, directly suppresses the proliferation and effector functions of cytotoxic and helper T-cells, further crippling the adaptive immune response. GIF is a well- characterized virulence factor of ORFV, which has been demonstrated in sheep [118,119].

Furthermore, ORFV severely impairs the host’s chemotactic gradients, which are essential to guide leukocytes to infection sites [120,121]. This disruption is mediated by the early immunomodulatory gene ORFV112, which encodes a secreted chemokine-binding protein (CBP). Although initially identified as a high-affinity inhibitor of CC-chemokines, structural studies demonstrated that CBP broadly sequesters chemokines across the C, CC, and CXC subfamilies. Crystallography of CBP bound to CCL2, CCL3, and CCL7 reveals that this broad-spectrum binding relies on its β-sheet II surface [122,123]. By neutralizing these chemokines, CBP prevents the recruitment of neutrophils, macrophages, and DCs to infected skin, thereby delaying viral clearance [124,125]. Consequently, CBP acts as a significant virulence factor contributing to the pathogenesis of ORFV in sheep [119,126].

#### Inhibition of NF-κB pathway

**Figure 3.**
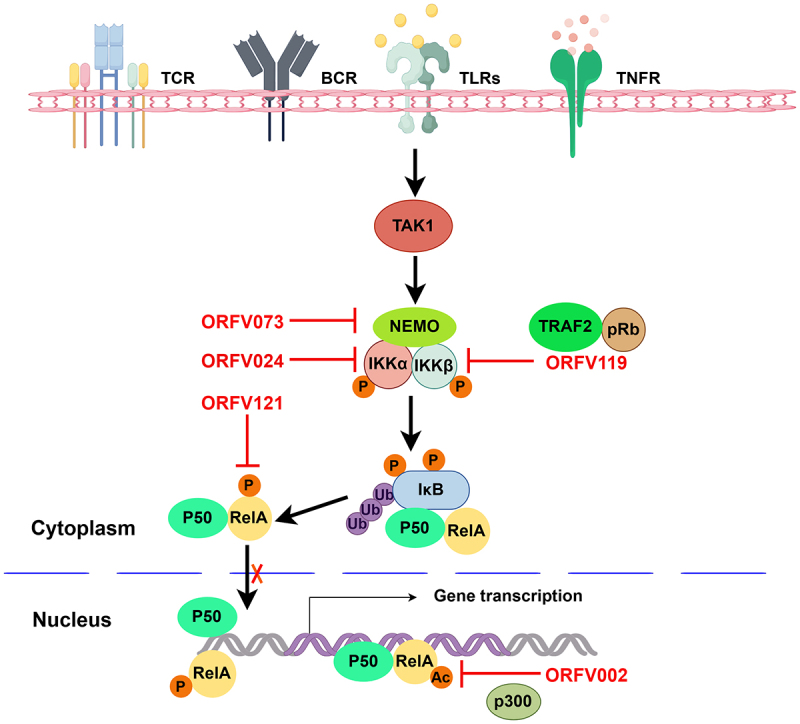
ORFV employs multiple viral proteins to inhibit NF-κB signaling pathway. Key mechanisms include: (1) ORFV002: binds NF-κB-p65 to block p300 acetylation and interacts with S100A4; (2) ORFV024: targets the IKK complex to suppress downstream signaling; (3) ORFV121: binds cytoplasmic p65 to block nuclear translocation; (4) ORFV119: links pRb/TRAF2 to inhibit IKK activation; (5) ORFV073: binds NEMO to prevent IKK activation. Most inhibitors are non-essential for in vitro replication but modulate host inflammation and/or virulence.

The transcription factor NF-κB is a master regulator of inflammation, controlling the expression of numerous pro-inflammatory mediators, including cytokines, chemokines, and adhesion molecules [127,128]. It is typically held inactive in the cytoplasm by its inhibitor, IκBα. Upon stimulation (e.g. by PRR signaling), the IKK complex phosphorylates IκBα, targeting it for degradation and allowing NF-κB to translocate to the nucleus. ORFV has evolved at least five distinct inhibitors of the NF-κB pathway, each targeting a different node of the signaling cascade (Figure 3, Table 1). These inhibitors differ in their subcellular localization, expression timing, and molecular targets, yet collectively ensure suppression of NF-κB-dependent inflammatory gene expression throughout the viral replication cycle.

**Table 1.** Summary of key ORFV immunomodulatory proteins and their roles in host-virus interactions.

Protein	Expression timing	Targeted pathway	Key mechanism of action	Virulence impact in natural host	References
ORFV020	Early	IFN signaling; PKR	Inhibits RIG-I signaling; suppresses PKR via dsRNA/PACT/ADAR1 interactions.	Not validated	[93,97–103]
ORFV057	Not defined	JAK/STAT pathway	Implicated in suppression of JAK/STAT signaling and ISG expression.	Not validated	[95]
ORFV116	Not defined	IFN signaling; NF-κB signaling	Antagonizes interferon and inflammatory responses.	Not validated	[96]
vIL-10 (ORFV127)	Early	Cytokines; Antigen presentation	Mimics host IL-10; suppresses pro-inflammatory cytokines and DC function.	Virulence factor	[108–115]
GIF (ORFV117)	Intermediate-late	Cytokines	Neutralizes GM-CSF and IL-2, impairing APCs and T cell activation.	Virulence factor	[116–119]
CBP (ORFV112)	Early	Chemokines	Sequesters CC, CXC, and C chemokines to prevent leukocyte recruitment.	Virulence factor	[120–126]
ORFV002	Early-late	NF-κB signaling	Blocks the phosphorylation and acetylation of the p65 subunit in the nucleus.	Non-virulence factor	[134–136]
ORFV024	Early	NF-κB signaling	Targets IKK complex, preventing IKKα/β phosphorylation.	Non-virulence factor	[131,132]
ORFV121	Early-late	NF-κB signaling	Binds p65 and blocks its phosphorylation and nuclear translocation.	Virulence factor	[133]
ORFV119	Early	NF-κB signaling; Apoptosis	Inhibits IKK activation via pRb/TRAF2; activates both intrinsic and extrinsic apoptosis.	Virulence factor	[130,145]
ORFV073	Early	NF-κB signaling	Targets NEMO to prevent IKK activation very early in infection.	Virulence factor	[129]
ORFV125	Not defined	Apoptosis	Viral Bcl-2 homolog that inhibits Bax/Bak-dependent apoptosis.	Not validated	[140–144]
ORFV129	Not defined	Complement system	Interacts with host C1QBP to suppress pro-inflammatory cytokines.	Not validated	[146]
VEGF-E (ORFV132)	Not defined	Angiogenesis	Binds VEGFR-2 and neuropilin-1 to promote vascular permeability and epidermal regeneration.	Virulence factor	[64–69]
PACR (ORFV014)	Not defined	Cell cycle	Mimics APC11 to inhibit APC/C complex, deregulating host cell cycle.	Not validated	[151,152]

APCs, antigen-presenting cells; DC, dendritic cell; CBP, chemokine-binding protein; GIF, GM-CSF and IL-2 inhibitory factor; VEGF-E, viral vascular endothelial growth factor E; vIL-10, viral interleukin-10; IKK, IκB kinase; ISG, interferon-stimulated gene; JAK/STAT, Janus kinase/signal transducer and activator of transcription; NEMO, NF-κB essential modulator; NF-κB, nuclear factor kappa B; PACR, poxvirus APC/C regulator; pRb, retinoblastoma protein; TRAF2, TNF receptor-associated factor 2.

At the very start of infection, ORFV targets the upstream IKK complex to halt the signal cascade early on. The virion-associated protein ORFV073 inhibits the pathway by targeting NEMO, thereby preventing IKK complex activation. Despite being non-essential for viral replication *in vitro*, ORFV073 is a key virulence determinant for ORFV in its natural host sheep [129]. Concurrently, another early-expressed virion-associated protein, ORFV119, interacts with the host tumor suppressor protein pRb via its LxCxE motif [130]. This ORFV119-pRb complex facilitates interaction with TRAF2, further blocking IKK complex activation. Following these immediate-early events, the early gene product ORFV024 continues to suppress upstream signaling by targeting the IKK complex directly, preventing IKKα/β phosphorylation [131]. Interestingly, while crucial for dampening inflammatory responses *in vitro*, ORFV024 does not affect viral virulence in sheep. Interaction of ORFV024 with host LAGE3 provides a crucial starting point for unraveling its specific modulatory mechanisms [132].

Further downstream, ORFV encodes proteins that directly interfere with the p65 subunit to prevent gene transactivation. The early-late protein ORFV121 functions by binding to cytoplasmic NF-κB-p65, blocking its phosphorylation and nuclear translocation. Crucially, ORFV121 acts as a major viral virulence factor, as its deletion significantly attenuates disease severity in sheep [133]. Even if p65 manages to reach the nucleus, ORFV exerts a final layer of control through the early-late protein ORFV002. ORFV002 localizes predominantly to the host cell nucleus, where it binds directly to NF-κB-p65 and disrupts its interaction with the acetyltransferase p300. This disruption prevents p300-dependent acetylation of NF-κB-p65 and consequently reduces the transactivating potential of NF-κB signaling. Furthermore, ORFV002 is non-essential for viral replication and virulence [134]. ORFV002 acts as a potent NF-κB signaling inhibitor by blocking MSK1-mediated phosphorylation of NF-κB p65 at Ser^276^ [135]. This blockade prevents the subsequent acetylation of p65 and impairs p300 recruitment, ultimately suppressing NF-κB-mediated gene transactivation. In addition, ORFV002 interacts with ovine S100A4, triggering its nuclear translocation, which is essential for ORFV002 to inhibit the NF-κB signaling pathway [136].

#### Modulation of apoptosis

Apoptosis is an essential cellular clearance mechanism operating via two main cascades. The extrinsic pathway is triggered by external death signals binding to cell surface death receptors, leading to activation of initiator caspases (e.g. caspase-8, −10). Conversely, the intrinsic (mitochondrial) pathway, is activated by internal stressors (e.g. DNA damage), causing mitochondrial outer membrane permeabilization and cytochrome c release, which then forms the apoptosome and activates caspase-9. Both pathways converge on executioner caspases such as caspase-3 and −7, which dismantle the cell by cleaving key proteins. This results in characteristic morphological changes including cell shrinkage, nuclear fragmentation, and formation of apoptotic bodies, which are phagocytosed by neighboring cells without inducing inflammation [137–139].

To ensure the infected host cell remains a viable factory for viral replication, ORFV encodes ORFV125, a mitochondrial protein that functions as a potent Bcl-2-like apoptosis inhibitor. ORFV125 prevents the activation of pro-apoptotic Bax and Bak, thereby blocking cytochrome c release and downstream apoptotic events [140]. ORFV125 directly binds and neutralizes a specific subset of pro-apoptotic BH3-only proteins (Bik, Puma, Bim isoforms, Noxa, DP5). In addition, it binds specifically to activated Bax, inhibiting its oligomerization [141]. This combined action provides significant anti-apoptotic protection for the virus. Crystallographic studies demonstrate that ORFV125 functions as a domain-swapped dimer that selectively binds pro-apoptotic BH3 motifs (notably Bax, Bak, Puma, Hrk) via its canonical Bcl-2 groove, undergoing a significant conformational change upon Bax binding, thereby elucidating the structural mechanism of viral apoptosis inhibition [142]. The crystal structures of ORFV125 bound to Puma and Hrk BH3 peptides provide the detailed structural basis for how this viral Bcl-2 protein selectively inhibits key host pro-apoptotic BH3-only proteins [143]. Tian et al. successfully identify five host proteins interacting with ORFV125, with BIRC5 being a particularly significant interactor due to its anti-apoptotic, pro-proliferative, and mitotic roles [144].

**Figure 4.**
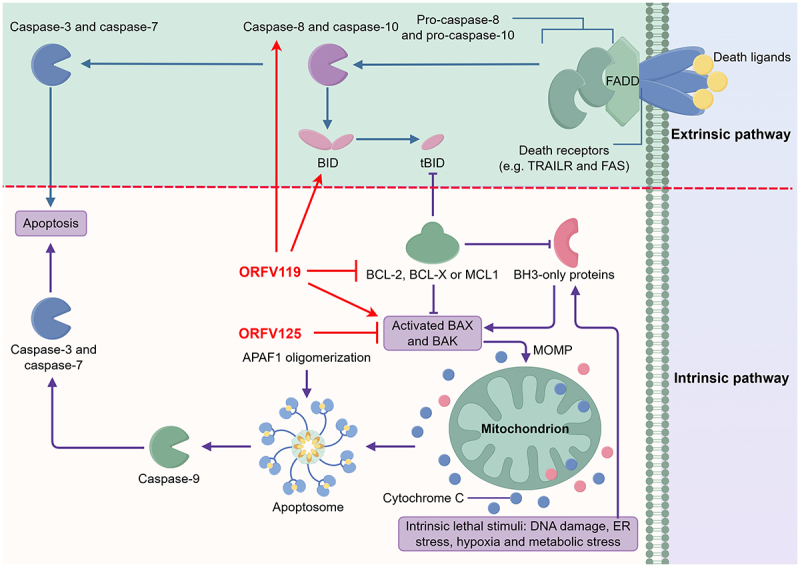
ORFV employs a dual strategy to modulate host cell apoptosis.

ORFV119 is a novel pro-apoptotic protein encoded by ORFV. It induces host cell apoptosis by simultaneously activating both the extrinsic (death receptor) and intrinsic (mitochondrial) pathways [145]. This is achieved through its mitochondrial localization, modulation of Bcl-2 family protein balance (increasing pro-apoptotic Bax/Bak/Smac, decreasing anti-apoptotic Bcl-2/cIAP-2), and activation of key caspases (8, 9, 3) leading to PARP cleavage. In conclusion, ORFV employs a dual strategy to modulate host cell apoptosis (Figure 4).

The virus both inhibits and induces apoptosis to optimize infection. The mitochondrial protein ORFV125 acts as a potent viral Bcl-2 homolog, directly binding and neutralizing pro-apoptotic proteins like Bax, Bak, and key BH3-only proteins (e.g. Bik, Puma) to prevent cytochrome c release and block intrinsic apoptosis. Conversely, ORFV119 is a pro-apoptotic viral protein that simultaneously activates both the intrinsic and extrinsic pathways by modulating Bcl-2 family balance and activating caspases 8/9/3.

#### Evasion of the complement system

A recent study demonstrated that ORFV129 interacts with host complement C1q binding protein (C1QBP) to modulate immune responses in goat turbinate bone cells [146]. While C1QBP itself has antiviral activity against ORFV, the ORFV129-C1QBP interaction contributes to ORFV immune evasion by suppressing key pro-inflammatory cytokines (IL-6, IL-1β, IFN-γ), likely through distinct signaling pathways.

### Evasion of adaptive immunity

#### ORFV suppresses MHC-I expression

If the innate response is breached, the adaptive immune system mounts a specific attack. ORFV has co-evolved mechanisms to counter this as well. For an effective T-cell response, viral antigens must be presented on major histocompatibility complex (MHC) molecules. ORFV actively suppresses MHC-I expression on the surface of infected cells. This makes the infected cells invisible to cytotoxic CD8^+^ T-cells, the primary killers of virus-infected cells [147]. As discussed above, the combined effects of vIL-10 and the GM-CSF/IL-2 inhibitor severely impair the function of DCs, the most potent antigen-presenting cells. They fail to mature properly, express co-stimulatory molecules, or migrate to lymph nodes, thus failing to initiate a robust T-cell response.

#### Antibody-dependent enhancement

The most recent study demonstrated that ORFV utilized antibody-dependent enhancement (ADE) to facilitate infection in both goat PBMCs and lip epithelial cells via distinct receptor systems: Fc receptors in immune cells and complement factor and complement receptors in epithelial cells [148]. This dual ADE strategy may enhance ORFV infection of diverse cell types and contribute to immune evasion. Notably, these ADE findings were obtained exclusively from *in vitro* systems, and whether ADE occurs *in vivo* at physiologically relevant antibody concentrations remains unknown. If confirmed in natural hosts, ADE could have important implications for vaccine safety, particularly for subunit or inactivated candidates that may elicit sub-neutralizing antibodies. However, the long-standing field use of live-attenuated ORFV vaccines without reported ADE-related adverse effects provides some reassurance. Further *in vivo* studies are needed to evaluate the biological relevance of ORFV ADE and its potential impact on vaccine design.

## Other cell processes regulated by ORFV

### Autophagy induction

The functional role of ORFV-induced autophagy in replication is complex. One early study in primary OFTu cells showed that while infection triggers autophagy, this process may not be essential for efficient viral replication [149]. Conversely, a recent study by the same team has demonstrated that ORFV induces autophagy in OFTU cells through dual modulation of the PI3K/AKT/mTOR and ERK1/2/mTOR pathways, and that this induction provides a pro-viral function, enhancing viral replication *in vitro* [150].

### Cell cycle manipulation

APC/C (Anaphase-promoting complex/cyclosome) is a large E3 ubiquitin ligase complex consisting of at least 12 subunits. Its catalytic core is formed by two specific proteins: APC2, a large scaffold protein, and APC11, a catalytic protein harboring a RING-H2 domain. Although it is essential for mitotic exit, its inactivation is required for the G1/S transition. PACR (poxvirus APC/C regulator) encoded by the ORFV014 gene, acts as an APC11 mimic by competing for binding to the APC2 scaffold. Because PACR can be incorporated into the APC/C complex but lacks ubiquitin ligase activity, it acts as a dominant-negative inhibitor of APC/C, thereby perturbing host cell cycle and facilitating viral replication [151,152].

### Oncolytic potential of ORFV

ORFV exerts a potent and selective anti-tumor effect against nasopharyngeal carcinoma (NPC) by inhibiting the mTOR signaling pathway, which subsequently triggers autophagy and ultimately leads to apoptosis of cancer cells. This specificity for cancer cells and favorable safety profile *in vivo* positions ORFV as a promising preclinical candidate for NPC treatment [153]. ORFV exerts significant oncolytic effects against colorectal cancer *in vitro* and *in vivo* by inhibiting tumor growth/metastasis, modulating key cytokines, and inducing apoptosis, highlighting its therapeutic potential [154]. ORFV exerts potent oncolytic effects against lung cancer cells by inducing caspase-dependent apoptosis and G2/M cell cycle arrest [155]. Crucially, it triggers immunogenic cell death (ICD), characterized by the release of DAMPs. This ICD, combined with enhanced CXCL16 secretion by infected tumor cells, promotes dendritic cell maturation and activates a robust antitumor immune response, particularly by recruiting and activating cytotoxic CD8^+^ T cells via the CXCL16/CXCR6 axis.

## Conclusions and future perspectives

The pathogenesis of ORFV is defined by its targeted infection of skin epithelia, rapid cytoplasmic replication, and the induction of characteristic proliferative lesions. While typically self-limiting, ORFV’s success as a pathogen is intrinsically linked to its many immunomodulatory proteins (Table 1) that orchestrate a multi-faceted evasion of host immunity. These mechanisms, ranging from IFN antagonism and NF-κB inhibition to the suppression of apoptosis and manipulation of chemokine gradients, allow the virus to establish a productive infection niche.

Despite significant advances, several key questions remain. It is important to acknowledge that the experimental systems used to characterize ORFV immunomodulatory mechanisms vary considerably, and this may influence the interpretation of findings. Several key virulence factors, including ORFV132, ORFV121, ORFV073, ORFV119, ORFV112, ORFV127, and ORFV117, have been validated in sheep using gene-deletion mutant viruses, providing direct evidence of their roles in natural host pathogenesis. In contrast, the mechanistic details of IFN antagonism (ORFV020, ORFV057, ORFV116) have been primarily characterized in human-derived surrogate cell lines such as HeLa, HEK293, and THP-1 cells. While these systems offer valuable mechanistic insights, species-specific differences in PRR repertoires, STING signaling, NF-κB regulation, and Fc receptor expression between human cell lines and ovine/caprine cells may affect the magnitude or even the nature of the observed responses. Future studies should prioritize the validation of key mechanistic findings in primary cells derived from natural hosts and in relevant *in vivo* infection models to ensure their biological significance. Furthermore, the functional redundancies and potential synergies between the numerous ORFV-encoded immune evasion genes are not fully understood. Although ORFV is highly adept at suppressing immune responses, harnessing it as an oncolytic vector requires striking a balance between that suppression and the activation of antitumor immunity-a delicate equilibrium that still needs fine-tuning.

Future research directions include further characterization of viral entry receptors, detailed analysis of how viral proteins manipulate specific signaling pathways, and investigation of host genetic factors that influence susceptibility and disease severity. Understanding these aspects will not only advance our knowledge of ORFV biology but also provide broader insights into epithelial immunity, wound healing, and the balance between immune protection and immunopathology. As we continue to uncover the mechanisms underlying ORFV pathogenesis and immune evasion, we gain valuable perspectives on the fundamental principles governing host-pathogen interactions and the evolutionary arms race between viruses and their hosts.

Omics-based research has greatly advanced our understanding of virus-host interactions, providing new insights into viral pathogenesis and host defense. However, comprehensive multi-omics datasets for ORFV, particularly from natural hosts, remain lacking. Additionally, functional validation is essential to establish the biological relevance of omics-derived findings. Future efforts should expand omics profiling across different host species and infection conditions, and integrate metabolomics, single-cell omics and spatial transcriptomics to resolve cell-type-specific responses.

## Data Availability

Data availability is not applicable to this review article as no new data were created.
